# Bomb blast injuries: an exploration of patient characteristics and outcome using Pakistan National Emergency Departments Surveillance (Pak-NEDS) data

**DOI:** 10.1186/1471-227X-15-S2-S7

**Published:** 2015-12-11

**Authors:** Irum Qamar Khan, Nadeem Ullah Khan, Rubaba Naeem, Salima Kerai, Kate Allen, Nukhba Zia, Sana Shahbaz, Shiraz Qayoom Afridi, Emaduddin Siddiqui, Uzma Rahim Khan, Adnan A Hyder, Junaid A Razzak

**Affiliations:** 1 Department of Emergency Medicine, Aga Khan Hospital, Karachi, Pakistan; 2 Johns Hopkins International Injury Research Unit, Department of International Health, Johns Hopkins Bloomberg School of Public Health, Baltimore, Maryland, USA; 3 Department of Emergency Medicine, Lady Reading Hospital, Peshawar, Pakistan; 4 Department of Emergency Medicine, Johns Hopkins School of Medicine, Baltimore, Maryland, USA; 5 The author was affiliated with the Department of Emergency Medicine, Aga Khan University, Karachi, Pakistan at the time when study was conducted

**Keywords:** injuries, bomb blast, surveillance, Pakistan

## Abstract

**Background:**

Bomb blast injuries result in premature deaths and burdening of healthcare systems. The objective of this study was to explore the characteristics and outcome of patients presenting to the emergency departments in Pakistan with bomb blast injuries.

**Methods:**

Active surveillance was conducted in seven major emergency departments of Pakistan from November 2010-March 2011. All the sites are tertiary care urban centers. All the patients who presented to the hospital's emergency department (ED) following a bomb blast injury as per self-report or the ambulance personnel were included in the study. Frequency of demographics, injury pattern, and outcomes were calculated.

**Results:**

A total of 103 patients with bomb blast injuries presented to the selected emergency departments. The median age of patients was 30 years. Around three-fourth of the patients were males (n = 74, 74.7%). Most of the bomb blast patients were seen in Peshawar (n = 41, 39.8%) and Karachi city (n = 31, 30.1%) and the most common mode of arrival was non-ambulance transport (n = 71, 76.3%). Upper limb injuries (n = 12, 40%) were common in the under 18 age group and lower limb injuries (n = 31, 39.2%) in the 18 years and above group. There were a total of 8 (7.7%) deaths reported out of these 103 patients.

**Conclusion:**

Bomb blast injuries in Pakistan generally affect young males. Non-ambulance transport is the most common way to access emergency departments (ED). Overall ED mortality is high and capturing data during a disaster in an emergency department is challenging.

## Background

Globally, 1.6 million lives are lost every year due to violence, 90% of which are in low- and middle-income countries (LMICs)[1,2]. For each death due to violence, there are dozens of hospitalizations, hundreds of emergency department visits, and thousands of outpatient visits [3]. Violence, at the individual or community level, is a violation of human rights and it often has lifelong consequences for victims' physical and mental health [3,4]. The effects of violence extend far beyond a family unit and have damaging effects at the community and national level [5-7].

The World Health Organization (WHO) classifies violence into three major groups: self-directed, interpersonal, and collective violence [8]. Of these, collective violence in the form of bomb blasts has become the most common form of violence [9]. Evidence suggests that damage in terms of lives lost, injuries, and infrastructure damage is substantial in blasts and explosions [10]. Bomb blasts also receive a high degree of public attention because of their significant effects on health in terms of death, illness, disability, and mental illness [1]. Blast injuries not only lead to premature deaths but consume more health care resources as compared to other injuries because they present in masses [7,11]. Moreover, youths are most affected with more severe injuries involving multiple body organs and systems [11,12].

According to a report between 2002-2009, civilians accounted for two-thirds of the suicidal terrorism-related deaths in Pakistan [13]. In a case series analysis from 2007-2011 of medico-legal death autopsies from three major public hospitals in Karachi, Pakistan revealed that there was a steady rise in the incidence of bomb blast injuries with the highest number of injuries reported in 2010 [14]. Among these cases, 95% were males between 15-45 years of age [14].

The objective of the paper is to report the frequency of bomb blast injuries and to describe the demographic and injury characteristics and outcomes of patients treated at major emergency departments in Pakistan.

## Methods

Pakistan National Emergency Departments Surveillance (Pak-NEDS) was a pilot active surveillance conducted in seven major emergency departments of Pakistan: Aga Khan University (AKU) and Jinnah Post-graduate Medical Center in Karachi; Benazir Bhutto Hospital in Rawalpindi; Lady Reading Hospital in Peshawar; Mayo Hospital in Lahore; Civil Hospital in Quetta; and Shifa International Hospital (SIH) in Islamabad. All the sites are tertiary care urban centers. AKU and SIH are private hospitals while the rest are public hospitals. AKU was the main coordinating center for the study. The study was reviewed and approved by the Institutional Review Boards of AKU and the Johns Hopkins Bloomberg School of Public Health. It was also given clearance by the similar ethical approval committees from each of the participating sites.

The data was collected between November 2010 and March 2011 at different time intervals for a period of 4 months in each participating site. A one-page standardized tool was developed to collect data. This tool was based on both the ambulatory care survey tool of the Centers for Disease Control and Prevention, USA and previous surveillance work conducted in Pakistan [15,16]. The Pak-NEDS data collection tool contained questions on patient demographics (age, gender), mode of arrival, body region injured, nature of injuries, and ED disposition. Individual identifying information was not collected from any of the study subjects. Confidentiality and anonymity were ensured for all individuals throughout the study. All electronic information was encrypted and password-protected.

Data collectors were specifically hired and trained for this study and worked in three shifts providing twenty-four hour coverage. All data collectors involved in Pak-NEDS were required to undergo training on the study questionnaire and all relevant data collection procedures including ethical conduct during the data collection process.

All the patients who presented to the hospitals' emergency departments (ED) following a bomb blast injury as per self-report or ambulance personnel were included in the study. Data collection was done through patient/next of kin interviews and reviews of the emergency department records. Waiver for consent was taken as no patient identifiers were collected. The on-site supervisors were responsible for sending the completed study forms to AKU (coordinating center) on a weekly basis. AKU was responsible for aggregating and safekeeping the study data, including the physical forms which are catalogued and kept in storage under lock and key in the department of Emergency Medicine at AKU. Injury causes and presenting complaints were coded using predefined coding lists. Data was entered at AKU using EpiInfo version 3.3.2 and analysis was done using SPSS version 20 [17,18] Proportions and percentages were calculated for categorical variables such as gender, mode of arrival, etc. Comparison of bomb blast injury characteristics and outcome was done for children (under 18 years) and adults (≥18 years) using Chi square test and Fisher Exact test with level of significance set at 0.05.

## Results

A total of 103 patients presented to the EDs of the seven major tertiary care hospitals in Pakistan. Of these, 7.7% (n = 8) died during their ED stay. The median age of patients was 30 years (interquartile range 25 - 75 years) based on self-reported data. About three-fourths of the patients were males (n = 74, 74.7%). Most of the patients were young adults of 26-45 years of age (n = 39, 37.9%). Table 1 gives patient demographic characteristics. About one-quarter of the patients were brought to the ED by ambulance (n = 22, 23.7%). Ambulance transport was a more common means (in 57% of cases) for access in Karachi city compared to other cities. According to the data no bomb blast patients went to the two private hospitals during the study time frame. Most of the bomb blast patients were seen in Peshawar (n = 41, 39.8%) and Karachi (n = 31, 30.1%).

**Table 1:** Demographic characteristics of bomb blast injury patients (n = 103)

Variables	n (%)
Gender*	
Males	74 (74.7)
Females	25 (25.3)

**Age groups in years**	
Up to 12	14 (13.6)
13 - 18	13 (12.6)
19 - 25	18 (17.5)
26 - 45	39 (37.9)
>45	19 (18.4)

Mode of arrival**	
Non Ambulance	71 (76.3)
Ambulance	22 (23.7)

**Hospitals**^†^	
LRH, Peshawar	41 (39.8)
AKU & JPMC, Karachi	31 (30.1)
MHL, Lahore	23 (22.3)
BBH, Rawalpindi	6 (5.8)
CHQ, Quetta	2 (1.9)
SIH, Islamabad	0 (0)

*Data on 4 participants were missing on gender

**Data on 10 participants were missing

^† ^Aga Khan University (AKU)

Jinnah Post-graduate Medical Center (JPMC)

Benazir Bhutto Hospital (BBH)

Lady Reading Hospital (LRH)

Mayo Hospital (MHL)

Civil Hospital (CHQ)

Shifa International Hospital (SIH)

There were differences in injury patterns between children (<18 years, n = 25, 24.3%) and adults (≥18 years, n = 78, 75.7%). In the under 18 years group, a majority had upper limb injuries (n = 12, 40%) followed by lower limb injuries (n = 7, 23%). In patients 18 years and above, most had lower limb injuries (n = 31, 39.2%) followed by head and neck injuries (n = 16, 20.2%). The majority of patients (92% of children and 63% of adults) were treated and discharged from the emergency department. Among children, only one was admitted to the hospital and one died. The child who died sustained injuries mostly in the chest, head, and neck region. Among adults, about one-quarter (n = 18, 25.4%) were admitted to the hospital and seven died. Table 2 gives a comparison of clinical characteristics of the patients under 18 years and those 18 years and above. However, no statistically significant differences were seen in body region injured, nature of injuries, and ED disposition when these clinical characteristics were compared with respect to age.

**Table 2:** Clinical characteristics of bomb blast injury patients (n = 103)

Variables	Under 18 years n = 25		18 years and above n = 78		p-value*
		
	n	**(%)**^†^	n	**(%)**^†^	
**Body region injured**					0.105
Head and neck	6	(20.0)	16	(20.2)	
Chest and abdomen	1	(3.0)	8	(10.1)	
Upper limbs	12	(40.0)	15	(18.9)	
Lower limbs	7	(23.0)	31	(39.2)	
Others	4	(13.0)	9	(11.3)	

**Nature of injuries**					0.471
Soft tissue injuries	18	(90.0)	34	(79.0)	
Fractures	2	(10.0)	7	(16.3)	
Contusion	0	(0)	2	(4.7)	

**ED Disposition**					0.069
Discharged from ED	22	(91.7)	45	(63.4)	
Admitted	1	(4.2)	18	(25.4)	
Died	1	(4.2)	7	(9.9)	
Others**	0	(0)	1	(1.4)	

† These are multi-response variable; denominator includes number of cases on whom data is available

*alpha is 0.05

** Left without being seen and left against medical advice

## Discussion

This study highlights the characteristics of patients with bomb blast injuries who were able to utilize ED services at the seven included major tertiary care centers with level 1 trauma care from all four provinces of the country during the study period. The study highlights that young males were most commonly affected, and that limb injuries were the most commonly affected body region in bomb blast incidents. Most of the bomb blast patients were seen in Peshawar, followed by Karachi city. Non-ambulance transport is the most common way to access EDs.

The finding that young males were the most common type of victims for bomb blast injuries is consistent with previous studies done in Pakistan [19]. One of the reasons behind this finding is that terrorists target large gatherings such as processions [20] and crowded places which are generally attended by young males and culturally avoided by females and the elderly [14].

In the literature, ED utilization rates varies based on bombing types, with the highest for confined space (97%), followed closely by open air (94%), and the lowest for structural collapse (48%) [21]. Bomb blast injuries in Pakistan are most often reported to be due to open air blasts, based on the pattern of injuries in a previous study [14]. We also found that among those who died, the pattern of injuries to body regions was consistent with open air type bomb injuries. Our study also showed that the most often affected body parts are the extremities. This is consistent with a study conducted in Karachi, Pakistan in 2013 [23].

The ED death rate in this study is 7.7% overall and 9.9% for adults 18 years and above. Since we only studied patients coming to the emergency department, this mortality rate does not include deaths that occurred at the scene or those who died after their admission to the hospital. The ED death rates reported in the literature for different types of bombing are close to zero, while immediate mortality ranges from 4%-25%, and mortality of those admitted to the hospital ranges from 15%-36% (21). Suicide bombing is the most common cause of blasts in Pakistan. These bombings are planned for maximum loss of lives and therefore mortality rates among bombing victims in Pakistan are likely to be higher than in bombings caused by accidental blasts or those not targeting crowded areas [21].

Bomb blasts present an acute burden on an already stretched emergency care system in Pakistan. Only one-quarter of the victims of bomb blasts in our study presented by ambulance. A lack of trained human resources and poor coordination between different stakeholders such as ambulances, law enforcement, and hospitals results in chaos [22,23]. Capacity-building of frontline healthcare providers in the acute management of bomb blast injuries should be given due attention. This can be done through short intensive courses which can be made mandatory for the ambulance and emergency department staff. Ensuring crowd control at the hospitals by law enforcement agencies is also necessary for the healthcare providers to provide lifesaving care without concern for their own safety. Similarly, citywide coordination led by city administration is important. Disaster management principles such as securing the scene, field triage, distribution of patients according to the capacity of the hospital, and - when required - secondary transfers to specialty centers would all be possible only through central coordination.

## Limitations

There are several limitations in this analysis. First, there may have been many injuries that were not captured in this study. This is especially relevant for bomb blasts where the sudden influx of patients and their relatives into EDs creates a chaotic environment making it difficult to effectively gather data. Secondly, the dataset did not contain information about the circumstances and locations of events. Thirdly, there is no information on the type of explosive devices involved. Such information would help us understand the mechanism of injuries caused by bomb blasts. Additionally, injury severity was not assessed. We suspect the coverage of Pak-NEDS and the data collectors' ability to capture all cases during sudden influxes of patients following a bomb blast are some of the reasons for poor capture.

The patterns of injuries, the outcome of injuries for those who make it alive to the hospital, and the care processes are some of the areas in which this ED surveillance study has been able to provide data. It is important to note that the EDs of the public hospitals that were study sites for Pak-NEDS are the main hospitals where all bomb blast patients are taken, which is why no bomb blast patients were recorded at the two private hospitals.

## Conclusion

Bomb blast injuries are common in Pakistan and affect the civilian population. Young males are mostly affected, with unexpectedly high ED mortality rates compared to reported mortality from other settings. Non-ambulance transport was much more frequent than ambulance transport. Additionally, low patient capture rates appear to be a challenge in data collection during a disaster. Future work should focus on developing, implementing, and evaluating integrated emergency plans for responding to such events.

## Competing interests

The authors declare that they have no competing interests.

## Authors' contributions

IQK, NUK, RN, SK, NZ, SS, EUS and URK were involved in initial draft development. NUK, RN, SK, NZ and URK were involved in data analysis and interpretation. KA, SQA, AAH and JAR provided critical review of the draft. AAH and JAR conceptualized Pak-NEDS and were involved in overall implementation of Pak-NEDS
